# No Correlation between PNPLA3 rs738409 Genotype and Fatty Liver and Hepatic Cirrhosis in Japanese Patients with HCV

**DOI:** 10.1371/journal.pone.0081312

**Published:** 2013-12-11

**Authors:** Masato Nakamura, Tatsuo Kanda, Shingo Nakamoto, Tatsuo Miyamura, Xia Jiang, Shuang Wu, Osamu Yokosuka

**Affiliations:** 1 Department of Gastroenterology and Nephrology, Chiba University, Graduate School of Medicine, Chiba, Japan; 2 Department of Molecular Virology, Chiba University, Graduate School of Medicine, Chiba, Japan; Saint Louis University, United States of America

## Abstract

**Background:**

Hepatitis C virus (HCV) infection is associated with the development of cirrhosis and hepatocellular carcinoma and is also related to fatty change of the liver. Variation in patatin-like phospholipase domain-containing 3 (PNPLA3) gene is associated with disease progression in nonalcoholic fatty liver disease (NAFLD). Recent reports have suggested that PNPLA3, IL28B and TLR4-associated single nucleotide polymorphisms (SNPs) may have an impact on hepatic steatosis or fibrosis in patients with chronic HCV infection.

**Methods and Findings:**

Four SNPs (PNPLA3 rs738409, TLR4 rs4986790, TLR4 rs4986791, IL28B rs8099917) were identified in Japanese patients infected with HCV. We examined the association between the distribution of these SNP alleles and fatty change of the liver or existence of hepatic cirrhosis diagnosed by ultrasonography, one of the widely accessible and easy-to-use methods. PNPLA3 rs738409 G-allele and IL28B rs 8099917 minor allele were found in 70.0% and 31.1%, respectively. These two TLR4 SNPs were uniform in Japanese. Fatty change of the liver developed independent of the abscence of hepatic cirrhosis on sonographic findings and younger age. Hepatic cirrhosis was associated with a higher aspartate aminotransferase/platelet ratio index (APRI), no fatty change of the liver, higher BMI and higher AFP levels. No association between PNPLA3 rs738409/IL28B rs8099917 genotypes and hepatic steatosis or liver fibrosis was observed.

**Conclusions:**

According to ultrasound examinations, no association between PNPLA3 rs738409 genotype and fatty change of the liver or hepatic cirrhosis was found in Japanese patients infected with HCV. Together, our results suggested that the mechanism of hepatic steatosis underlying HCV infection might differ from that of NAFLD and should be explored.

## Introduction

It is estimated that hepatitis C virus (HCV) infection affects approximately 170 million people worldwide [1], [2]. Chronic hepatitis C infection is associated with the development of cirrhosis and hepatocellular carcinoma [3], [4]. The association of steatosis with chronic hepatitis C has been well described and has shown to occur in up to 66% of cases [5], [6]. Steatosis accelerates activities and progression of chronic hepatitis C and is independently associated with stage III/IV hepatic fibrosis [5]. Overall sustained virological response to treatment in HCV-infected patients with steatosis is also considerably lower than in those without steatosis [6].

Studies of the mechanism of steatosis in chronic hepatitis C are limited. It was reported that HCV core protein induced hepatic steatosis in transgenic mice [7]. Nishina et al. [8] observed fat accumulation in the liver in transgenic mice expressing HCV polyprotein and reported that iron-induced unfolded protein response appeared to be one of the mechanisms responsible for hepatic steatosis in HCV infection. HCV particles were observed in close proximity to lipid droplets, an organelle used for the storage of neutral lipids that moves dynamically through the cytoplasm, interacting with other organelles, including the endoplasmic reticulum [9]. These findings indicate that some steps of HCV assembly take place around lipid droplets [9], suggestng that this might be possible mechanism for HCV directly inducing hepatic steatosis.

Patatin-like phospholipase domain-containing 3 (PNPLA3), which encodes a 481 amino acid protein, is a triacylglycerol lipase conserved from potatoes to humans with 10-fold higher expression in liver compared to adipose tissue [10]. Variation in PNPLA3 gene contributes to ethnic and inter-individual differences in hepatic fat content and susceptibility to nonalcoholic fatty liver disease (NAFLD) [11]. It was reported that the minor allele of rs738409 C/G, a nonsynonymous coding single nucleotide polymorphism (SNP) in the PNPLA3 gene encoding I148M change, was associated with steatosis, portal inflammation, lobular inflammation, Mallory-Denk bodies, NAFLD activity score (NAS) and fibrosis [12]. The minor allele of rs738409 C/G is also strongly associated with hepatic fat content and with elevated serum levels of ALT and AST [11]–[15].

Toll-like receptor 4 (TLR4) is a receptor for bacterial lipopolysaccharide (LPS), which is suggested to be involved in the pathogenesis of hepatobiliary diseases [16]–[18]. It was reported that the minor allele of rs4986791 c.1196C>T, a nonsynonymous coding SNP in the TLR4 gene encoding T399I change, emerged as conferring protection against fibrosis progression compared to a major, wild-type (WT) CC allele, along with another highly consegregated SNP (rs4986790, c.896A>G) located at codon position 299 (p.D299G) [19]. These TLR4 SNPs have been related to a blunted response to LPS and to susceptibility to infectious diseases and sepsis [20], [21], and they are associated with protection against hepatic fibrosis, reduce TLR4-mediated inflammatory and fibrogenic signaling, and lower the apoptotic threshold of activated hepatic stellate cells [22]. TLR4 SNPs also modulate the risk of liver fibrosis in Caucasians with chronic hepatitis C infection [23].

There were several reports that SNP located upstream of the interleukin-28B (IL28B) gene (rs8099917) was associated with the response to peginterferon-alpa plus ribavirin therapy in chronic hepatitis C patients [24], [25]. Although the data remain contradictory, associations of IL28B genotype (rs12979860) with hepatic steatosis and liver fibrosis were reported in previous studies [26], [27].

Therefore, we genotyped four SNPs (PNPLA3 rs738409, TLR4 rs4986790, TLR4 rs4986791 and IL28B rs8099917) in Japanese patients infected with HCV. We also compared the distribution of these SNP alleles with fatty change of the liver on ultrasonography (US) of those patients. In addition, we examined the association between the distribution of these SNP alleles and the existence of hepatic cirrhosis diagnosed by US in those patients. Our results suggest that the distribution of these SNP alleles does not have any significant impact on fatty change of the liver or the existence of cirrhosis revealed by US in Japanese patients with chronic hepatitis C.

## Results

### Baseline characteristics

Two hundred and sixty patients infected with HCV were enrolled in this study. Baseline characteristics of the patients are shown in Table 1. All patients were Japanese had a median age of 55.6 years, and 137 (52.7%) were male. Mean body mass index (BMI) was 23.1 kg/m^2^, with 45 and 9 of these patients classified as overweight (25.0–29.9 kg/m^2^) and obese (≥30 kg/m^2^), respectively. Only one patient with HCV genotype 3 was included. US revealed fatty change of the liver and hepatic cirrhosis in 127 (47.6%) and 37 (14.2%), respectively.

**Table 1:** Baseline characteristics of the 260 patients infected with HCV.

Age (years)	55.6±11.4
Gender (male/female)	137/123
Body mass index (kg/m^2^)	23.1±3.58
HCV genotype (1/others)	192/68
HCV RNA levels (high/low)	235/25
ALT (IU/L)	69.6±67.2
γ-GTP (IU/L)	49.3±52.7
Platelet counts (×10^4^/μL)	16.9±5.3
Total cholesterol (mg/dL)	175±30.1
AFP (ng/mL)	8.9±14.8
APRI	1.07±0.98
Fatty liver, yes/no	124/118
Cirrhosis, yes/no	37/223

± standard deviation. HCV RNA levels, high: ≥5 logIU/mL; HCV RNA levels, low: <5 logIU/mL; ALT, alanine aminotransferase; γ-GTP, gamma-glutamyl transpeptidase; APRI, aspartate aminotransferase/platelet ratio index: AST (IU/L)/35/PLT (10^3^/μL) ×100; fatty liver and hepatic cirrhosis were diagnosed by ultrasonography. Data are presented as mean

### Genotype frequencies of SNPs in PNPLA3, IL28B and TLR4 genes

**Figure 1:**
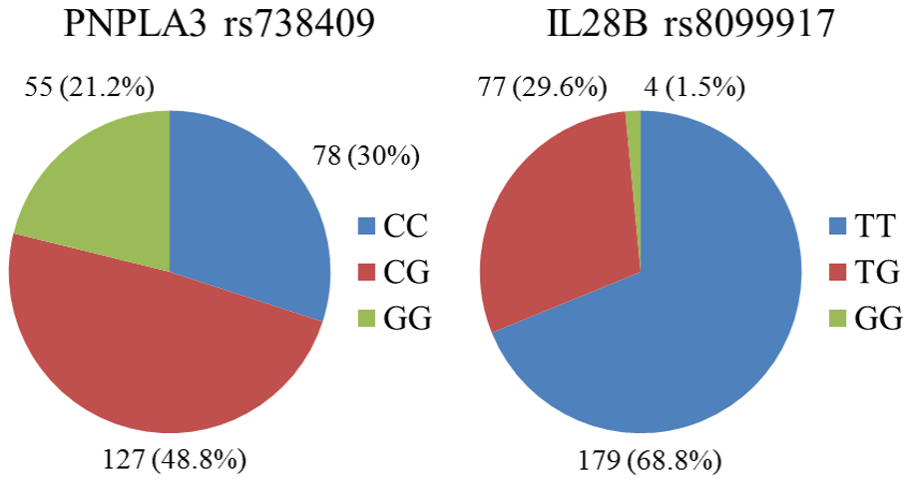
Distribution of single-nucleotide polymorphisms (SNPs) in patatin-like phospholipase domain-containing 3 (PNPLA3) and interleukin-28B (IL28B) genes in 260 HCV-infected patients.

Genotype frequencies of PNPLA3 rs738409 and IL28B rs8099917 genotypes among the patients are shown in Figure 1. Among the 260 total patients, 182 (70.0%) and 81 (31.1%) had PNPLA3 rs738409 G-allele and IL28B rs 8099917 G-allele, respectively. All patients were AA genotype of TLR4 rs4986790 and CC genotype of TLR4 rs4986791. These results indicated that the Japanese patients in the present study had uniform distribution of these two TLR4 SNPs.

### No association between PNPLA3 rs738409 genotype and fatty change of the liver or existence of hepatic cirrhosis diagnosed by US

Clinical characteristics of the patients in the present study were compared between the PNPLA3 rs738409 G-allele and non-G allele groups (Table 2). There were no significant differences between the two groups, although HCV RNA levels tended to be higher in the PNPLA3 rs738409 G-allele group than in the non-G allele group. US showed similar fatty change of the liver in the two groups as well as similar distribution of patients with advanced fibrosis in the present study (Table 2).

**Table 2:** Patient characteristics according to PNPLA3 rs738409 genotype.

Characteristics	G allele (n = 182)	Non-G allele (n = 78)	P-value*
Age (years)	55.7±11.4	55.4±11.3	0.845
Gender (male/female)	94/88	43/35	0.704
Body mass index (kg/m^2^)	23.06±3.4	23.3±3.9	0.678
HCV genotype (1/others)	130/52	62/16	0.229
HCV RNA levels (high/low)	168/14	67/11	0.168
IL28B rs8099917 (TT/TG+GG)	125/57	54/24	0.953
ALT (IU/L)	71.0±67.6	66.6±66.7	0.629
γ-GTP (IU/L)	51.0±54.8	45.2±47.7	0.417
Platelet counts (×10^4^/μL)	17.3±9.1	17.4±5.5	0.928
Total cholesterol (mg/dL)	175±29.5	175±31.6	1.000
AFP (ng/mL)	9.72±16.9	7.39±8.56	0.249
APRI	1.11±1.04	0.99±0.84	0.368
Fatty liver, yes/no	86/96	38/40	0.935
Cirrhosis, yes/no	25/157	12/66	0.876

± standard deviation. *P-value, between two groups with and without PNPLA3 rs738409 G allele by Student's t-test or chi-square test; HCV RNA levels, high: ≥5 logIU/mL; HCV RNA levels, low: <5 logIU/mL; ALT, alanine aminotransferase; γ-GTP, gamma-glutamyl transpeptidase; APRI, aspartate aminotransferase/platelet ratio index: AST (IU/L)/35/PLT (10^3^/μL) ×100; fatty liver and hepatic cirrhosis were diagnosed by ultrasonography. Data are presented as mean

### No association between IL28B rs8099917 genotype and fatty change of the liver or existence of hepatic cirrhosis diagnosed by US

The patient characteristics of those with IL28B rs809917 genotype are shown in Table 3. γ-GTP levels were higher in the IL28B rs809917 minor allele (TG or GG) group than in the major allele (TT) group. However, the present study did not show any association between IL28B rs809917 genotype and fatty change of the liver or the existence of hepatic cirrhosis based on US findings.

**Table 3:** Patient characteristics according to IL28B rs8099917 genotype.

Characteristics	TT (n = 179)	TG or GG (n = 81)	P-value*
Age (years)	56.1±11.4	54.5±11.4	0.295
Gender (male/female)	97/82	40/41	0.558
Body mass index (kg/m^2^)	22.8±3.5	23.6±3.7	0.094
HCV genotype (1/others)	127/52	65/16	0.153
HCV RNA levels (high/low)	162/17	73/8	0.895
PNPLA3 rs738409 (GG+GC/CC)	125/54	57/24	0.953
ALT (IU/L)	68.7±70.3	71.6±60.1	0.747
γ-GTP (IU/L)	43.0±39.9	63.4±72.1	<0.01
Platelet counts (×10^4^/μL)	20.1±25.2	16.9±5.7	0.260
Total cholesterol (mg/dL)	177±31.1	172±27.7	0.215
AFP (ng/mL)	8.18±15.8	11.0±11.8	0.152
APRI	1.03±0.97	1.16±1.01	0.324
Fatty liver, yes/no	82/97	42/39	0.441
Cirrhosis, yes/no	23/156	14/67	0.449

± standard deviation. *P-value, between two groups with IL28B rs8099917 TT and with TG/GG by Student's t-test or chi-square test; HCV RNA levels, high: ≥5 logIU/mL; HCV RNA levels, low: <5 logIU/mL; ALT, alanine aminotransferase; γ-GTP, gamma-glutamyl transpeptidase; APRI, aspartate aminotransferase/platelet ratio index: AST (IU/L)/35/PLT (10^3^/μL) ×100; fatty liver and hepatic cirrhosis were diagnosed by ultrasonography. Data are presented as mean

### Comparison of patient characteristics with and without fatty change of the liver

The patient characteristics with and without fatty change of the liver based on US finding results are shown in Table 4. By univariate analysis, younger age (P<0.01), higher BMI (P<0.01), and a lower proportion of cirrhotic patients (P<0.01) were observed in patients with fatty change of the liver. Factors significantly associated with fatty change of the liver by univariate analysis were analyzed by multivariate logistic regression analysis again (Table 5). Fatty change of the liver was attained independent of the abscence of hepatic cirrhosis on US findings and age ≤58 years.

**Table 4:** Comparison of patient characteristics with or without fatty change of the liver.

Characteristics	Fatty liver (+)	Fatty liver (−)	P-value*
Number	124	136	
Age (years)	52.7±11.6	58.2±10.6	<0.01
Gender (male/female)	62/62	75/61	0.480
Body mass index (kg/m^2^)	24.0±3.8	22.3±3.2	<0.01
HCV genotype (1/others)	87/37	105/21	0.250
HCV RNA levels (high/low)	110/14	125/11	0.500
ALT (IU/L)	75.6±80.4	64.2±52.3	0.172
γ-GTP (IU/L)	53.6±53.7	45.3±51.7	0.205
Platelet counts (×10^4^/μL)	19.4±20.1	18.8±22.3	0.820
Total cholesterol (mg/dL)	176±30.5	174±29.9	0.650
AFP (ng/mL)	9.1±14.3	8.89±15.2	0.908
APRI	1.02±0.96	1.12±1.00	0.908
Cirrhosis, yes/no	9/115	28/108	<0.01

± standard deviation. *P-value, between two groups with and without fatty liver by Student's t-test or chi-square test; HCV RNA levels, high: ≥5 logIU/mL; HCV RNA levels, low: <5 logIU/mL; ALT, alanine aminotransferase; γ-GTP, gamma-glutamyl transpeptidase; APRI, aspartate aminotransferase/platelet ratio index: AST (IU/L)/35/PLT (10^3^/μL) ×100; fatty liver and hepatic cirrhosis were diagnosed by ultrasonography. Data are presented as mean

**Table 5:** Factors associated with fatty change of the liver by multivariate analysis.

Factor	Category	Odds ratio	95% CI	P-value
Age (years)	58≤/<58	0.523	0.315–0.862	0.012
Cirrhosis	yes/no	0.328	0.146–0.734	0.006

Hepatic cirrhosis was diagnosed by ultrasonography.

### Comparison of patient characteristics with and without hepatic cirrhosis

The patient characteristics with and without hepatic cirrhosis according to US findings are shown in Table 6. By univariate analysis, higher BMI (P = 0.016), higher γ-GTP (P<0.01), lower platelet count (P = 0.046), lower total cholesterol (P = 0.013), higher AFP (P<0.01), higher aspartate aminotransferase/platelet ratio index (APRI) (P<0.01) and a lower proportion of fatty change of the liver (P<0.01) were observed in patients with hepatic cirrhosis. By multivariate logistic regression analysis, hepatic cirrhosis was attained independent of higher APRI, no fatty change of the liver on US findings, higher BMI and higher AFP levels (Table 7).

**Table 6:** Comparison of patient characteristics with or without hepatic cirrhosis.

Characteristics	Cirrhosis (+)	Cirrhosis (−)	P-value*
Number	37	233	
Age (years)	58.9±10.6	55.0±11.4	0.052
Gender (male/female)	22/15	115/108	0.476
Body mass index (kg/m^2^)	24.3±2.9	22.8±3.6	0.016
HCV genotype (1/others)	31/6	145/78	0.038
HCV RNA levels (high/low)	32/5	203/20	0.570
ALT (IU/L)	81.2±46.9	67.6±70.0	0.255
γ-GTP (IU/L)	86.7±101.0	42.7±35.2	<0.01
Platelet count (×10^4^/μL)	12.7±5.4	20.2±22.7	0.046
Total cholesterol (mg/dL)	164±26.6	177±30.3	0.013
AFP (ng/mL)	15.6±14.6	7.92±14.6	<0.01
APRI	1.78±1.02	0.96±0.93	<0.01
Fatty liver, yes/no	9/28	115/108	<0.01

± standard deviation. *P-value, between two groups with and without hepatic cirrhosis by Student's t-test or chi-square test; HCV RNA levels, high: ≥5 logIU/mL; HCV RNA levels, low: <5 logIU/mL; ALT, alanine aminotransferase; γ-GTP, gamma-glutamyl transpeptidase; APRI, aspartate aminotransferase/platelet ratio index: AST (IU/L)/35/PLT (10^3^/μL) ×100; fatty liver and hepatic cirrhosis were diagnosed by ultrasonography. Data are presented as mean

**Table 7:** Factors associated with hepatic cirrhosis by multivariate analysis.

Factor	Category	Odds ratio	95% CI	P-value
AFP (ng/mL)	6≤/<6	2.394	1.018–5.631	0.045
Body mass index (kg/m^2^)	23.5≤/<23.5	2.665	1.164–6.099	0.022
Fatty liver	yes/no	0.201	0.080–0.503	<0.01
APRI	1≤/<1	9.035	3.339–24.44	<0.01

^3^/μL) ×100; fatty liver was diagnosed by ultrasonography. APRI, aspartate aminotransferase/platelet ratio index: AST (IU/L)/35/PLT (10

## Discussion

In the present study, we used abdominal US to evaluate fatty change of the liver and the existence of hepatic cirrhosis in Japanese patients with chronic hepatitis C. Despite our study population being relatively small, PNPLA3 rs738409 and IL28B rs8099917 genotypes seemed to have no association with fatty liver or the presence of hepatic cirrhosis although TLR4 rs4986790 and rs4986791 genotypes were uniform in the patients of the present study.

Hepatic steatosis is frequently observed in chronic hepatitis C patients (42–73%) [28] and is influenced by several factors, such as alcohol comsumption, age, BMI, obesity, hyperglycemia, diabetes mellitus and HCV genotype 3 [29]. Hepatic steatosis in chronic hepatitis C patients is associated with more severe liver damage, more advanced liver fibrosis [30], and poor response to peginterferon-alpha plus ribavirin treatment [31], [32]. Despite the majority of our study patients being HCV genotype 1 or 2, fatty change of the liver was associated with younger age and absence of hepatic cirrhosis (Table 5).

It had been reported that PNPLA3 rs738409 was associated with hepatic steatosis and steatohepatitis in NAFLD [11], [33]. The GG genotype was shown to be related to a greater risk of inflammation and cirrhosis. Japanese studies [34]–[36] also showed similar results. Kitamoto et al. [36] demonstrated that Matteoni type4 NAFLD is both a genetically and clinically different subset from the other spectrums of the disease and that the PNPLA3 gene is strongly associated with the progression of nonalcoholic steatohepatitis (NASH) in Japanese, whose BMI is lower than that of the United States and European countries [37]–[39].

In chronic hepatitis C patients, several studies suggested that PNPLA3 genotype influences hepatic steatosis and liver fibrosis [40], [41]. However, our results showed no association between PNPLA3 rs738409 and hepatic steatosis or liver fibrosis. It is possible that the different evaluation methods, such as US and liver biopsy, and different ethnicity or different distribution of PNPLA3 rs738409 genotypes might have different association with hepatic steatosis and liver fibrosis in Japanese patients with chronic hepatitis C. We also could not completely rule out the possibility that there exist different mechanisms of hepatic steatosis and liver fibrosis between NAFLD and hepatitis C [7]–[9]. Further studies will be needed regarding this point.

In contrast to previous studies [26], [27], IL28B rs8099917 also did not influence hepatic steatosis and liver fibrosis in the present Japanese study. US is a widely accessible and easy-to-use diagnostic imaging modality suitable for the qualitative assessment of diffuse liver disease in clinical daily practice [42]. Clinically, steatosis or fatty liver appears as a brighter image relative to the adjacent kidney or spleen and shows greater attenuation in severe cases, often obscuring the hepatic and portal vein walls [42]. The difference in results between the previous studies and ours might be ascribed to the methods used for the diagnosis of fatty liver and hepatic cirrhosis [43]. Although liver biopsy remains a standard method of diagnosis of diffuse liver disease, it is certainly an invasive technique compared to US. In the near future, transient elastography will be useful for the evaluation of hepatic fibrosis in chronic hepatitis C patients with hepatic steatosis. However, at present, it should be interpreted cautiously in NAFLD patients, where host- or disease-related factors may modify its accuracy [44].

Hepatocellular carcinoma is a documented complication in an unknown percentage of cases of NASH cirrhosis [45]. Yoshioka et al. [46] reported that the noncancerous liver showed burn-out NASH; steatosis, necroinflammation, ballooning degeneration, and Mallory bodies had all disappeared at the autopsy of an HCC case with NASH cirrhosis. Of interest, our present study also demonstrated the association between fatty change of the liver and no advanced fibrosis in Japanese patients with chronic hepatitis C.

In conclusion, the present study demonstrated that there was no association between PNPLA3 rs738409 genotype/IL28B rs8099917 genotype and hepatic steatosis or liver fibrosis in Japanese patients infected with HCV, although the results of our study were based on US findings. We found that fatty change of the liver was associated with patient age and liver fibrosis. Additionally, hepatic cirrhosis was also associated with higher APRI, no fatty change of the liver, higher BMI and higher AFP levels. Greater accuracy of non-invasive methods for the diagnosis of patients might be desired to enable further analysis. Together, our results suggest that the mechanism that exists in hepatic steatosis of HCV infection might differ from that of NAFLD, and additional study will be needed to further explore these mechanisms.

## Materials and Methods

### Patients

A total of 260 patients with chronic hepatitis C were consecutively recruited for the present study at Chiba University School of Medicine Hospital, Chiba, Japan, between February 2010 and January 2013. The study protocol was approved by the Ethics Committee of Chiba University School of Medicine (permission numbers 244, 374 and 1462) and conformed to the ethical guidelines of the Helsinki Declaration. Written informed consent was obtained from all patients.

### HCV genotyping

HCV genotype was determined using the antibody-based HCV serotyping assay reported by Tsukiyama-Kohara et al. [47]. In Japan, more than 98% of HCV genotype 1 belongs to HCV subgenotype 1b [48], [49], according to Simmonds' classification [50].

### HCV RNA quantification

The HCV RNA level was determined using Amplicor HCV monitor assay, version 2.0 (Roche Diagnostics, Tokyo, Japan), or the COBAS TaqMan HCV test (Roche). We defined HCV RNA ≥5 log IU/mL and <5 log IU/mL as high and low viral titers, respectively.

### Characterization of SNPs

To prepare DNA samples from blood cells, we used DNA Extract All Lysis Reagents (Applied Biosystems Inc., Foster City, CA, USA). A specific TaqMan genotyping assay was performed for PNPLA3 rs738409, IL28B rs8099917, TLR4 rs4986790, or TLR4 rs4989791. All TaqMan probes were purchased from Applied Biosystems. PCR was performed with TaqMan GTXpress Master Mix (Applied Biosystems) according to the manufacturer's protocol. PCR conditions were as follows: 95°C for 20 seconds, followed by 40 cycles at 95°C for 3 seconds and 60°C for 20 seconds. Fluorescent signals were evaluated using the ABI Step One real-time PCR system (Applied Biosystems) [51], [52].

In the present study, PNPLA3 rs738409 GG/CG or CC allele was analyzed as G allele or non-G allele, respectively, and IL28B rs8099917 TT or TG/GG was also analyzed as major allele or minor allele, respectively.

### Hepatic fibrosis score

APRI: AST (IU/L)/35/PLT (10^3^/μL) ×100, was evaluated as hepatic fibrosis score [53].

### Ultrasound findings of fatty change of the liver and hepatic cirrhosis

We carried out transabdominal US for evaluation of fatty liver and cirrhosis at least twice when ruling out hepatocellular carcinoma [42], [43]. For hepatitis C patients, abdominal US was routinely performed every 4–6 moths in Japan. Fatty liver was diagnosed when seeing at least one of the following: (1) diffuse enhancement of echoic levels in the liver, (2) hepato-renal contrast, (3) gradual attenuation of echoic levels in the liver, (4) unclear display of hepatic blood vessels. Cirrhosis was diagnosed when all of the following criteria were observed: (1) round blunt border of the liver, (2) surface irregularity, (3) rough echoic appearance in the parenchyma of the liver, with or without splenomegaly or collateral vessels.

### Statistical analysis

Data are expressed as mean ± standard deviation (SD). Differences were evaluated by Student's t-test or chi-square test. *P<0.05* was considered statistically significant.
